# Sports injury and illness incidence at the 2021 Summer Universiade: a retrospective study of 6,500 athletes from 113 countries/regions

**DOI:** 10.3389/fpubh.2025.1532849

**Published:** 2025-04-30

**Authors:** Zhiwei Li, Shiyun Li, Zhigang Liu, Shunchang Li, Huang Huang, Tutu Wang, Geping Wang

**Affiliations:** ^1^School of Sports Training, Chengdu Sport University, Chengdu, China; ^2^Clinical Medical College & Affiliated Hospital, Chengdu University, Chengdu, China; ^3^Health Commission of Chengdu, Chengdu, China; ^4^Institute of Sports Medicine and Health, Chengdu Sport University, Chengdu, China

**Keywords:** sports medicine, epidemiology, illness, injury, competition

## Abstract

**Objective:**

To provide insights and recommendations for athletes, medical personnel, and event organizers on optimize healthcare services and preventive measures for injuries and illnesses by describing the pattern of injuries and illnesses sustained during the 2021 Summer World University Games.

**Methods:**

Medical records of athletes visiting Universiade Athletes’ Village Polyclinic from July 22 to August 10, 2023, were anonymously analyzed to describe the injuries and illnesses sustained.

**Results:**

A total of 478 athletes visited the clinic, including 315 injuries and 163 illnesses. Translating to 9.5 injury visits and 3.1illness visits per 100 athletes. A total of 4.9% of athletes experienced at least one injury, and 2.5% experienced at least one illness. The injuries involved 540 sites, primarily affecting the thighs, shins, knees, lower back, and ankles. Illnesses predominantly involved the respiratory system and digestive system. Thirteen cases of COVID-19, and single cases of H1N1 influenza, malaria, dengue fever, candida intestinal infection, and infectious mononucleosis were detected.

**Conclusion:**

Injuries and illnesses accounted for 7.4% of the participants. The injury incidence was lower compared to the Summer Olympics and slightly below similar Winter Universiade events, while the illness incidence was higher. Using the electronic medical record visit record data of the hospital information system in the athlete village to study athlete injuries is a method that can be adopted in future large-scale sports events. Taking appropriate infectious disease prevention and control measures can effectively prevent the prevalence of infectious diseases during international large-scale sports events. The athlete village of international large-scale sports events should be equipped with MRI to meet the diagnosis and treatment needs of athletes.

## 1 Introduction

Monitoring sports injuries and illnesses constitutes the initial step in injury and illness prevention (1, 2), crucial for safeguarding athletes’ health and ensuring their competitive readiness (3–7). Extended periods of high-intensity training and competition pose significant health risks to athletes, a primary cause for early termination of athletic careers (8–10). Particularly during major events, high level of attention was drawn to the injuries and illnesses among athletes (11–16). Epidemiological surveillance during these events provides vital insights into injury and illness rates within the sporting domain (11, 15, 17). According to Van Mechelen et al.’s four-stage model of injury epidemiology, gathering descriptive data is the foundational step for developing effective prevention strategies (18, 19). Therefore, understanding the characteristics of injuries and illnesses among elite athletes during major sporting events is crucial for mitigating and preventing the injuries and illnesses.

The World University Games, as one of the most significant global sporting events for university students, attract a large number of elite student athletes from different countries and regions. The 2021 Summer World University Games in Chengdu were postponed for 2 years due to the global COVID-19 pandemic. This was the first global sporting event hold after the lifting of restrictions on the novel coronavirus pneumonia that has had a devastating effect on the world, presenting a formidable challenge for all event organizers and medical personnel to safeguard athlete health, prevent epidemics of infectious diseases and ensure the successful hosting of the games. Currently, there is limited research on the epidemiological data of large-scale sports events among elite university athletes. To our knowledge, only one study has described the injury and illness situation during the 2015 Winter University Games (20), leaving a gap in research on the risks of injuries and illnesses among athletes in the Summer World University Games. Understanding and exploring the epidemiological characteristics of injuries and illnesses among athletes at the Summer World University Games is crucial for devising effective prevention and management measures.

The purpose of our study is to describe the characteristics of sports injuries and illnesses treated at the Athletes’ Village polyclinic during the 2021 Summer World University Games and to provide practical insights and recommendations for athletes, medical personnel, and event organizers to plan and optimize healthcare services and preventive measures for injuries and illnesses. In our knowledge, this is the first report to use hospital information system to research the injuries and illnesses of athletes in athletes’ village. Additionally, this paper analyzes the utilization of the Athletes’ Village polyclinic by athletes to offer insights for future medical support planning at the Summer World University Games.

## 2 Materials and methods

All patients requiring medical attention are required to attend the polyclinic, which is the sole facility within the athletes’ Village. The personal registration information of all the patients treated in the polyclinic were reviewed, in order to verify it with appointment information. The whole process of diagnosis and treatment in the polyclinic was systematically recorded in electronic medical records by uniformly trained doctors.

The identity of an athlete was determined based on the information of the person who registered at the 2021 Summer World University Game. The electronic medical records of athletes who visited the athletes’ village polyclinic from the opening to the closure of the Universiade Village, that is, from July 22, 2023 to August 10, 2023, were anonymized and retrieved, and the injury and illness of athletes at the polyclinic were analyzed, after the end of the 2021 Summer World University Games.

### 2.1 Outcomes

We recorded all injuries and illnesses that were new, persist (continuation of treatment for athletes injured in training for the Universiade before entering the Universiade Village) or recurring (athletes having returned to full participation after a previous condition) musculoskeletal complaints, concussions or other medical conditions (injuries) or illnesses incurred in competition or training during the period of the 2021 Summer World University Games (from July 22 to August 8, 2023) receiving medical attention, regardless of the consequences with respect to absence from competition or training (14).

Injury and illness were defined as per the International Olympic Committee consensus statement on methods for recording and reporting of epidemiological data on injury and illness in sport (21). That is, injury was defined as tissue damage or other derangement of normal physical function due to participation in sports, resulting from rapid or repetitive transfer of kinetic energy, and illness was defined as a complaint or disorder experienced by an athlete, not related to injury. Classifications of injuries by body area and by tissue and pathology type, and illnesses by organ system/region adhered to the International Olympic Committee consensus statement (21, 22).

### 2.2 Confidentiality and ethical approval

We treated all information confidentially, and deidentified our database after the Games, ensuring anonymity of all athletes. These data have not been used for any other purpose. The approval of the ethic committee of Affiliated Hospital of Chengdu University has been also obtained (SC-08-011).

### 2.3 Data analysis

We calculated the summary measures of injury and illness incidences (i) according to the formula i = n/e, where n is the number of injuries or illnesses in competition, training, or in total during the study period and the respective number of exposed (participating) athletes; with incidence proportions presented as injuries/illnesses per 100 athletes. We present injury and illness incidences as means and risk ratios with 95% confidence intervals. We regarded two-tailed *p* values <0.05 as significant.

## 3 Results

The 2021 Summer World University Games saw participation from 113 countries/regions and 6,500 athletes. The peak period for athlete visits to the polyclinic occurred on the fifth competition day, concentrated between 20:00 and 21:00. A total of 478 athletes sought medical treatment due to injuries or illnesses, primarily focusing on rehabilitation and sports medicine at the polyclinic. Among these 478 athletes (315 injured, 163 ill), the average age was 22.2 years (range 19 to 27). They collectively made 819 visits due to injuries or illnesses, which translates to 7.4 athletes per 100 experiencing at least one instance of injury or illness (95% CI 6.7 to 8.0), with an average of 12.6 visits per 100 athletes. Specifically, male athletes made 405 visits (237 athletes), while female athletes made 414 visits (241 athletes), resulting in an average of 1.7 visits per athlete (Table 1).

**Table 1:** Distribution of injuries and illnesses among visiting athletes.

Category	Male		Female		Total		
	Number	Visit	Number	Visit	Number	Visit	Injuries or illnesses per 100 athletes
Injury	171	323	144	293	315	616	9.5
Illness	66	82	97	121	163	203	3.1
Total	237	405	241	414	478	819	12.6

### 3.1 Temporal distribution of athletes’ medical visits

**Figure 1:**
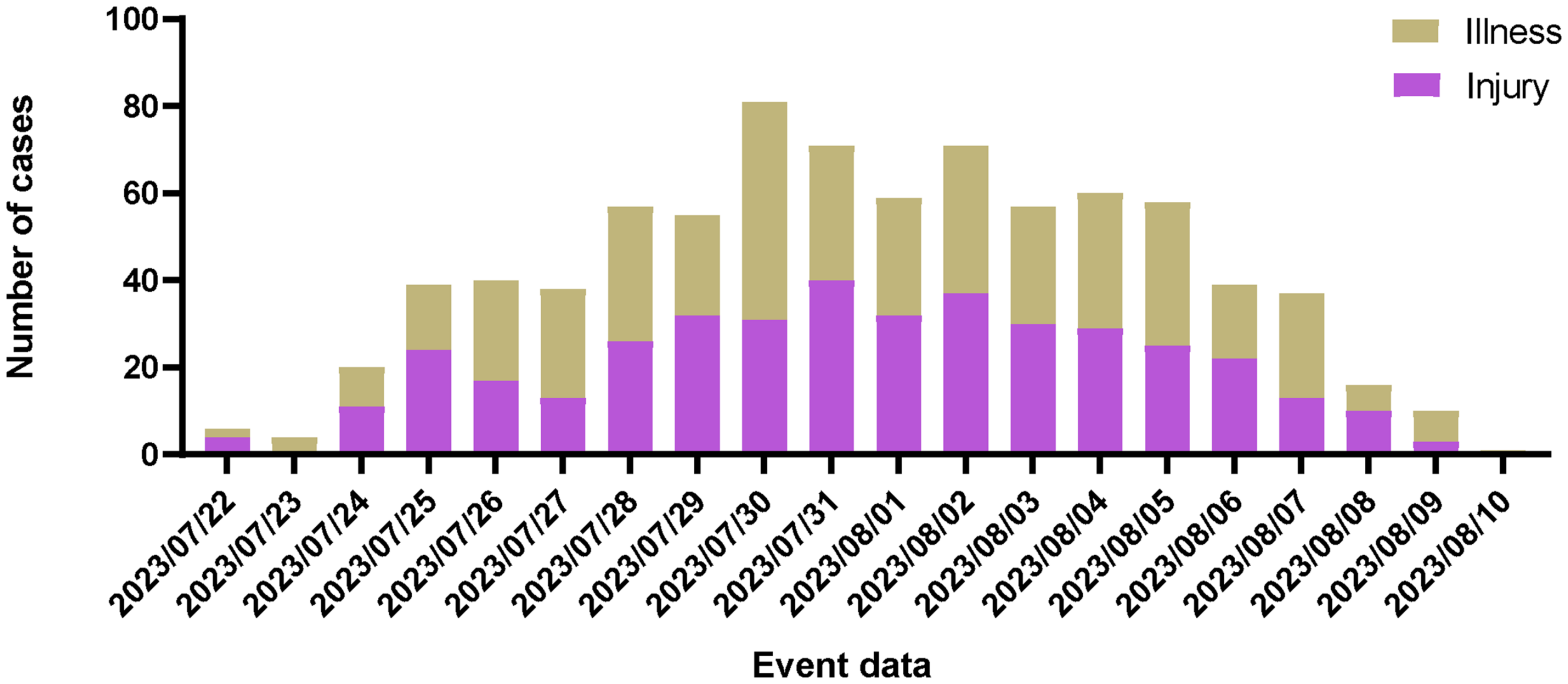
Distribution of daily clinic visits by athlete.

**Figure 2:**
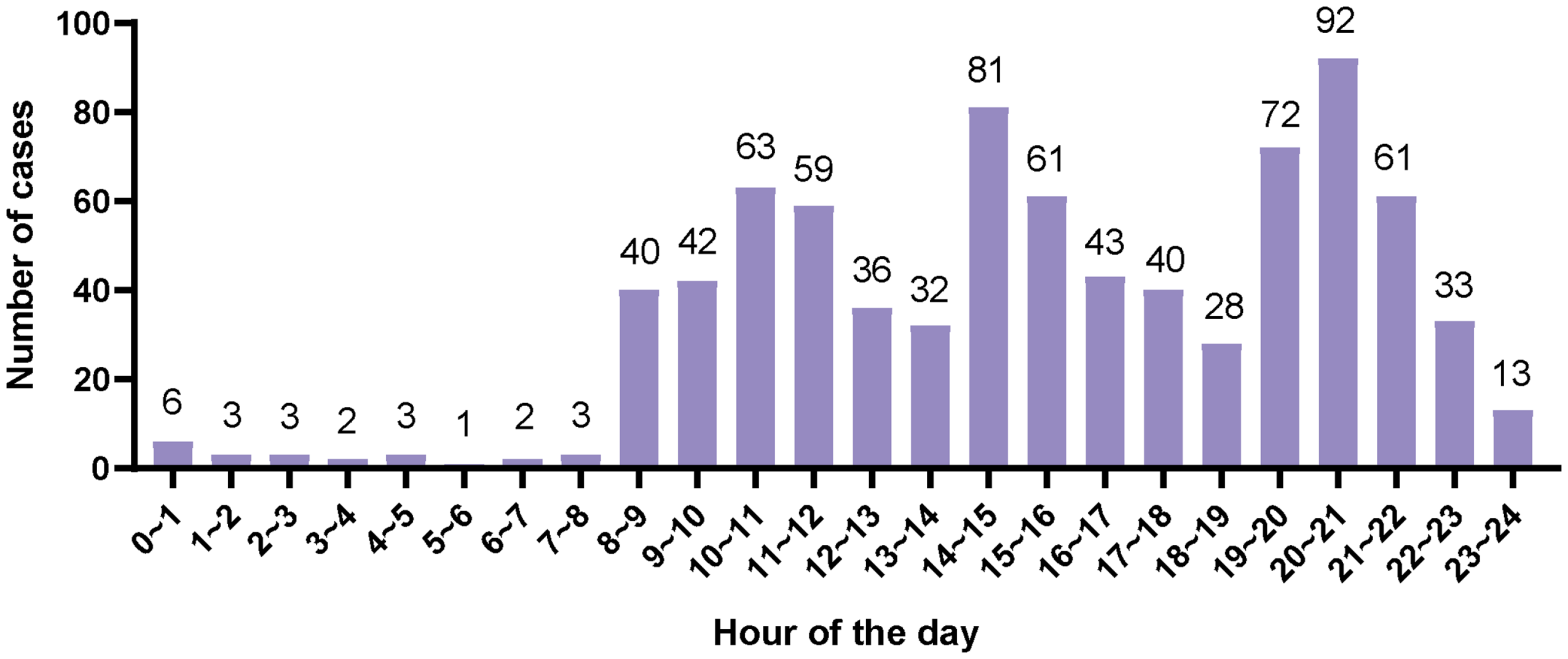
Distribution of athletes’ clinic visits by time periods throughout the day at the general clinic.

During the 2021 Summer World University Games, there was a general trend in athlete visits to the Athletes’ Village Clinic, starting low, peaking on the fifth competition day, and then tapering off (Figure 1). Throughout the day, visits to the clinic were predominantly concentrated between 8:00 and 23:00, with a peak in treatments occurring between 20:00 and 21:00 (Figure 2).

### 3.2 Distribution of athletes visited departments

**Figure 3:**
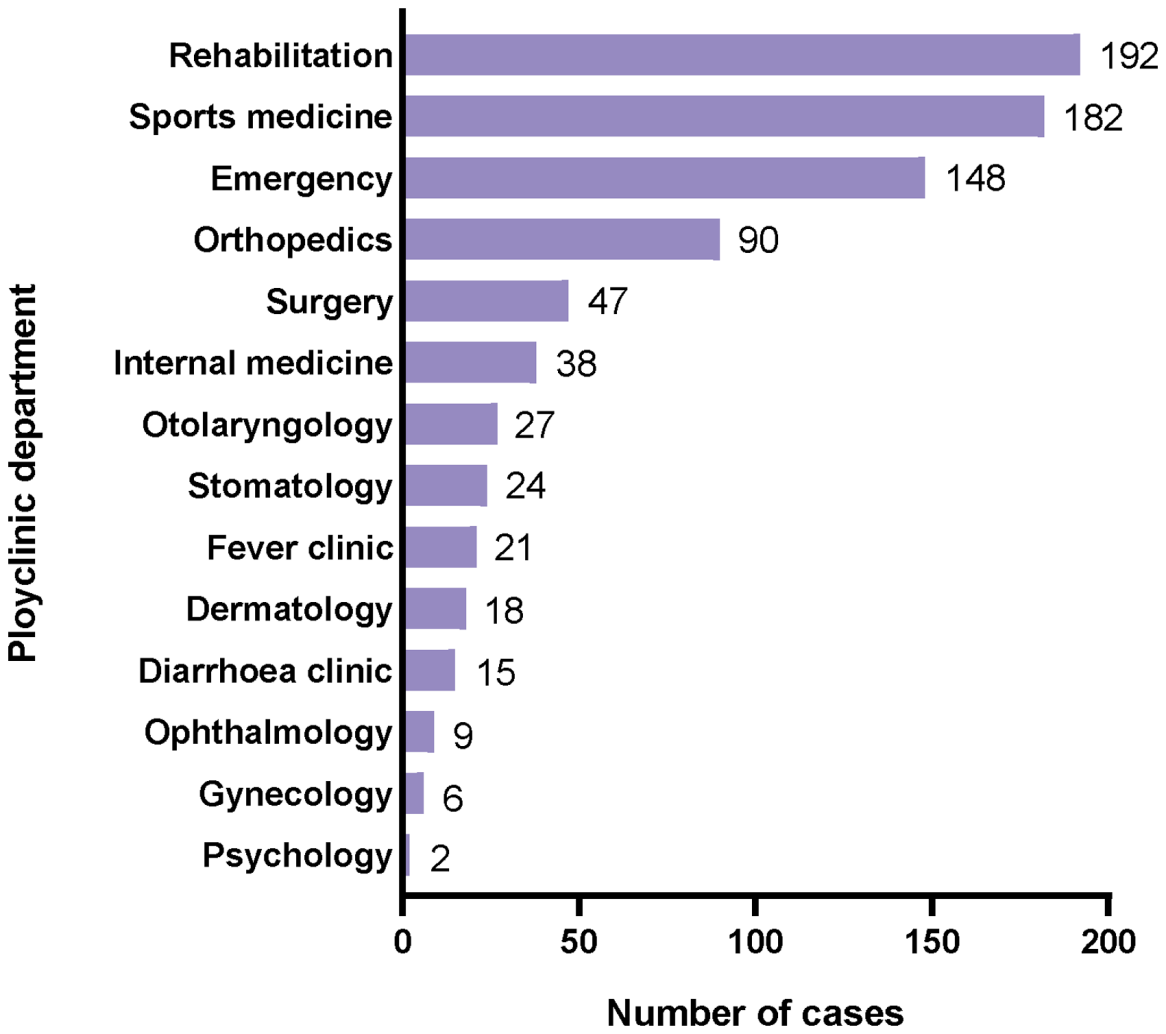
Distribution of athletes visited departments at the polyclinic.

Athletes primarily sought treatment in the departments of rehabilitation (*n* = 192), sports medicine (*n* = 182), orthopedics (*n* = 90), and surgery (*n* = 47), totaling 62.4% of all visits (Figure 3). Emergency visits accounted for 148 cases, constituting 18.1% of the total visits, including 46 cases for injuries and 102 for illnesses.

### 3.3 Examination and treatment of athletes

Athletes at the Athletes’ Village polyclinic underwent rehabilitation therapy (*n* = 229), medication treatments (*n* = 161), MRI scans (*n* = 78), CT scans (*n* = 41), laboratory tests (*n* = 38), and ultrasound examinations (*n* = 25). One athlete was transferred to an external designated hospital due to heatstroke from the polyclinic.

### 3.4 Athletes’ visits due to injury

315 athletes made 616 visits due to injuries, equivalent to 9.5 visits per 100 athletes. At least 4.9 athletes (95% CI 4.3 to 5.4) experienced one injury. The average number of visits per injured athlete was 2.0 times, with 41.6% (*n* = 131) of injured athletes visiting two or more times. Among injured male athletes, 171 individuals made 323 visits, averaging 1.9 visits per person, with 40.9% (*n* = 70) visiting two or more times. Among injured female athletes, 144 individuals made 293 visits, averaging 2.0 visits per person, with 42.4% (*n* = 61) visiting two or more times (Table 2).

**Table 2:** Distribution of athletes visited polyclinic due to injuries.

Category	Number	Visits	Average visits per person	Once visit	Twice or more visits	Percentage (%)
Male	171	323	1.9	101	70	40.9
Female	144	293	2.0	83	61	42.4
Total	315	616	2.0	184	131	41.6

### 3.5 Locations of athletes’ injuries

**Figure 4:**
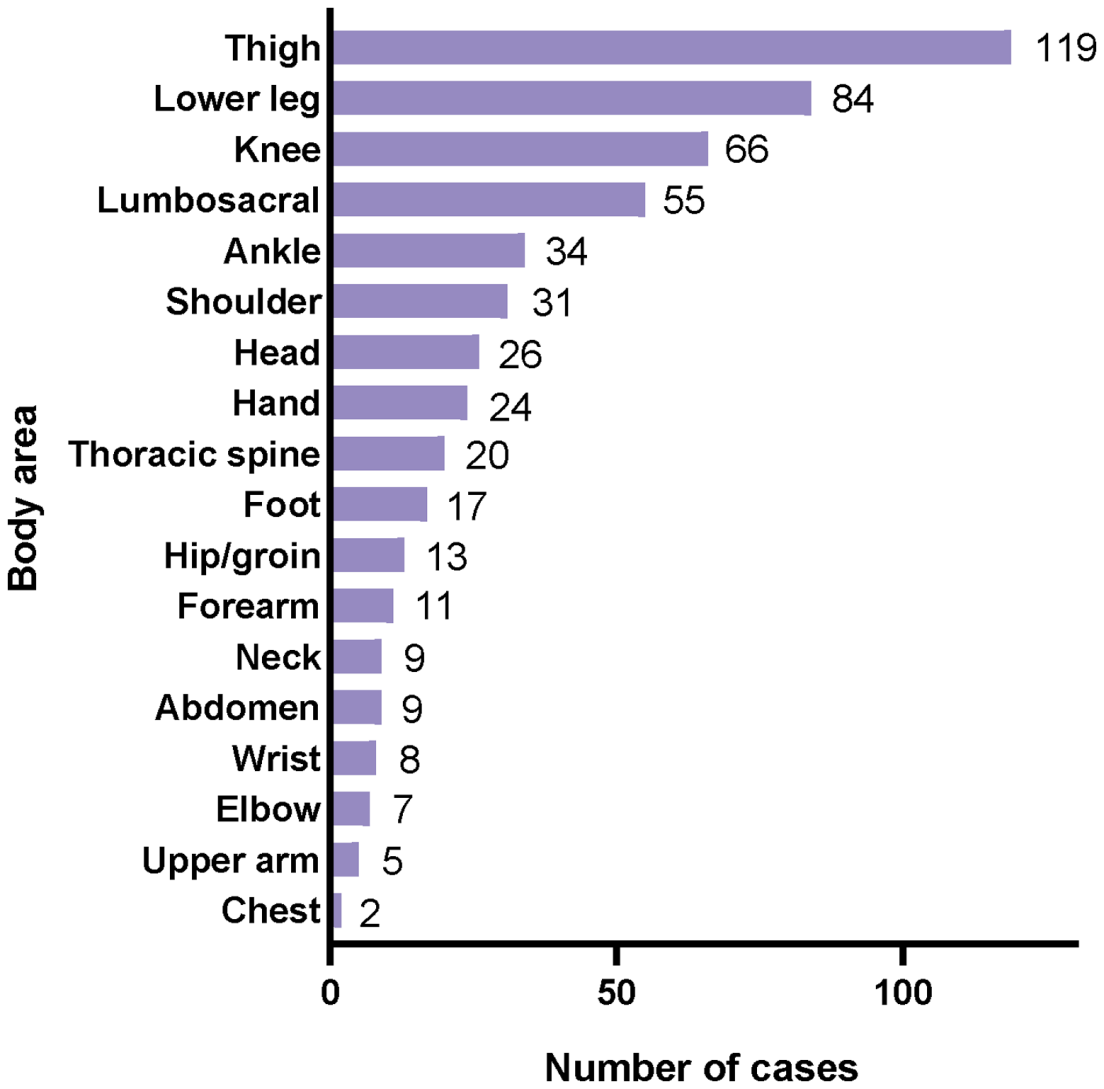
Distribution of injury locations of athletes visited the polyclinic.

The injuries of 315 athletes involved 540 anatomical sites, equivalent to 8.3 injured sites per 100 athletes, with an average of 1.7 sites per injured athlete. Among these, males had 307 affected sites with an average of 1.8 sites per injured individual, while females had 233 sites with an average of 1.6 sites per injured individual. The distribution of injury sites is depicted in the Figure 4, with the thigh being the most commonly injured at 22.0% (*n* = 119), followed by the calf at 15.6% (*n* = 84), the knee at 12.2% (*n* = 66), the lower back at 10.2% (*n* = 55), and the ankle at 6.3% (*n* = 34). Among injured athletes, 36.2% (*n* = 114) sustained injuries to two or more sites. Specifically, 38.0% of male athletes (*n* = 65) and 34.0% of female athletes (*n* = 49) sustained injuries to two or more sites.

### 3.6 Causes of athletes’ injuries

84.8% (*n* = 267) of injuries were related to training or competition, which means at least 4.1 athletes per 100 experienced an injury related to training or competition. This includes 248 new injuries, equating to 3.8 new injuries per 100 athletes, 13 cases of recurrent old injuries, and 6 cases of old injuries requiring ongoing treatment. Among these injuries, 45.1% (*n* = 142) occurred during competition and 39.7% (*n* = 125) during training. Additionally, 15.2% involved injuries unrelated to sports or training, including 41 new injuries as 16 cases of minor injuries whose causes cannot be determined in medical records, 15 cases of injuries caused accidentally during athletes’ outings for visits and celebrations, 6 cases of injuries related to facilities and roads in the athlete village, and 4 cases of bites and scratches by wild animals or insects in the village and 7 cases of recurrent old injuries.

### 3.7 Athletes visited polyclinic due to illness

**Figure 5:**
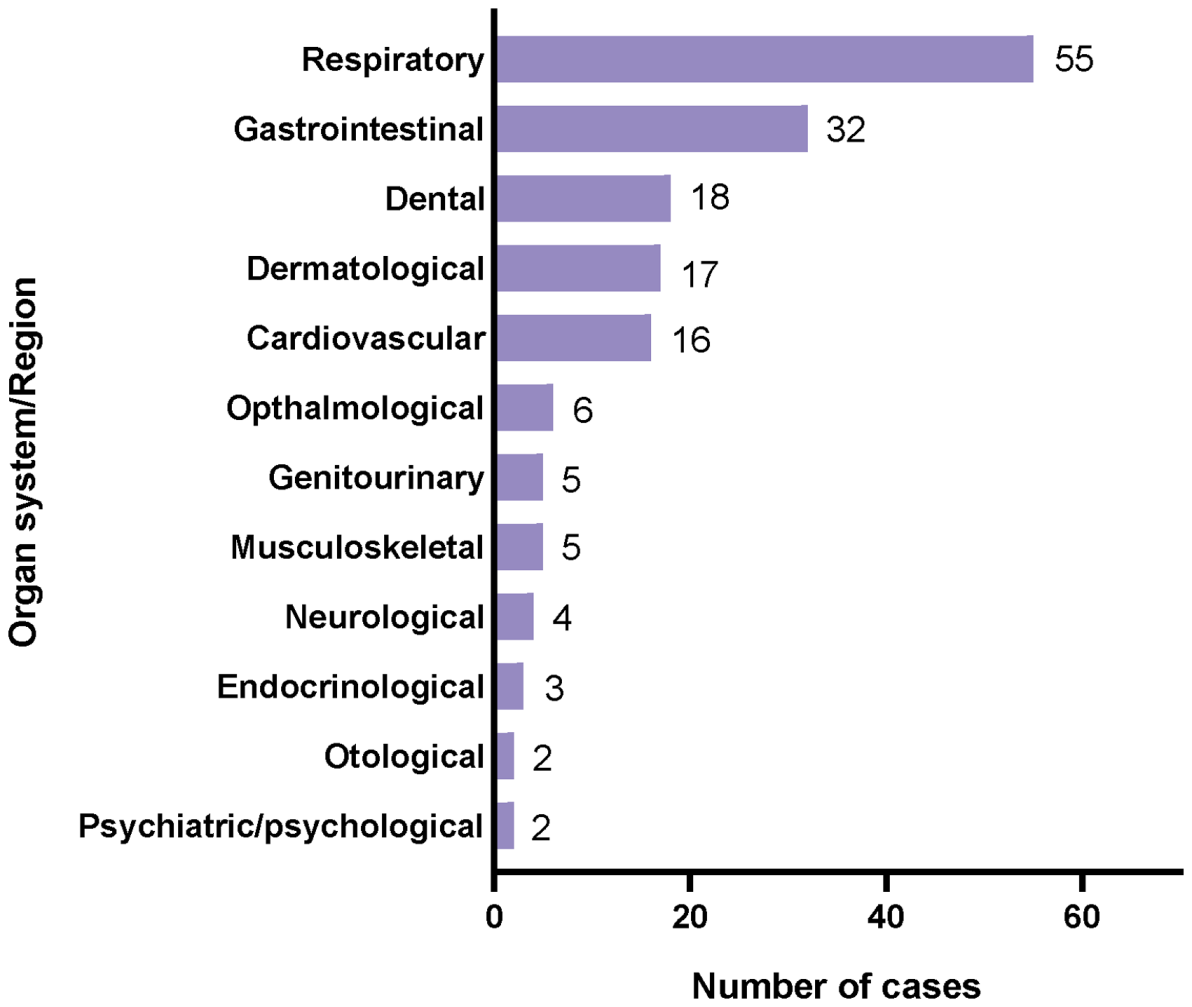
Distribution of athletes’ clinic visits due to illness.

163 athletes attended medical appointments 203 times due to illness, equating to 2.5 cases per 100 athletes (95% CI 2.1 to 2.9). Among them, 66 males attended 82 appointments, while 97 females attended 121 appointments. Respiratory system disorders (*n* = 55) and digestive system disorders (*n* = 32) were the most prevalent illnesses. The distribution of illnesses categorized by system is illustrated in the Figure 5.

No expected infection issues occurred during the 2021 Summer World University Games. Thirteen cases of novel coronavirus pneumonia were detected among athletes at the polyclinic, along with one case of H1N1 influenza virus infection, malaria, Kyasanur Forest disease, Cryptococcus intestinal infection, and infectious mononucleosis, respectively.

## 4 Discussion

To our knowledge, this study represents the first report on injuries and illnesses among athletes at the World University Summer Games. We conducted a retrospective case study using electronic medical records of athlete visited the polyclinic in the athlete village during the 2021 Summer World University Games. Major findings include: (1) Among 6,500 athletes, 4.9 and 2.5% experienced at least one injury or illness, with overall visit rates of 9.5 visits per 100 athletes for injuries and 3.1 visits per 100 athletes for illnesses. Athletes visited for injuries at approximately twice the rate of illnesses. (2) At least 4.1 athletes per 100 experienced an injury related to training or competition, with 45.1% of injured athletes sustaining injuries during competition and 39.7% during training. The most common injury sites were the thigh (22.0%), calf (15.6%), and knee (12.2%). (3) Respiratory (*n* = 55) and digestive (*n* = 32) systems were the most prevalent illnesses.

### 4.1 Athletes visited the village polyclinic

The athletes’ village guarantees the medical treatment of athletes with the help of a polyclinic converted from a hospital of Chengdu University. In accordance with the requirements of the International University Sports Federation for the Athletes’ Village Polyclinic of the Summer Universiade, the Polyclinic served the delegation for a total of 20 days from the opening day of the 2021 Summer World University Games to 2 days after the closing ceremony. The number of visits from the start of the tour on July 22 to July 28 was gradually increased with the number of people staying in the athletes’ village. The competition was held from July 27 to August 8, mainly from July 29 to August 7, during which there was a significant increase in the number of injuries due to athletes, and the peak of medical treatment occurred on the fifth day after the start of the competition, because the number of injury visits reached 40, and then the number of injury visits gradually decreased. Because the peak of medical treatment for the disease reached 50 on the fourth day after the start of the competition, this day was also the peak of medical treatment for athletes in this competition. The peak of the number of events in the 2021 Summer World University Games was on August 6, when the athletes were only half of their respective peaks in terms of trauma and illness. The number of visits by athletes gradually decreases from peak as the schedule progresses, similar to the polyclinic visits at other major international games. The distribution of time for athletes’ visits fluctuated significantly. There are three peaks of medical treatment, the highest peak is from 20 to 21 o’clock in the evening, followed by 14 to 15 o’clock and 10 to 12 o’clock, which may be related to the competition events during the daytime, and the trough of the number of medical visits is related to the service schedule of the polyclinic and the life and rest of athletes. Optimizing the deployment of healthcare workers by accounting for attendance variations across different times of the day and days during the Universiade can minimize reduce resource consumption while maintaining adequate medical care provision.

The emergency department was open 24 h a day, serving 148 athletes, of which only 1/3 were treated due to injury and illness, and 2/3 were treated with illness. Similar to other games, the demand for rehabilitation physiotherapy was much higher than that of medication (23). Although there was no official requirement for equipping MRI in the Polyclinic in the Summer Universiade, considering the experience of Olympic medical security and the dependence of sports injury diagnosis and treatment on MRI during the preparation of medical security for the athlete village, this Universiade has equipped MRI in the Polyclinic of the athlete village and provided diagnostic services for 78 people. Therefore, it is necessary to equip MRI in the athlete village for large-scale sports events including the Universiade.

### 4.2 Injury risk during the world university games

At the 2021 Summer World University Games, 315 athletes sought medical attention 616 times due to injuries and illnesses, averaging 9.5 visits per 100 athletes. At least 4.9% of athletes sustained injuries during the Games, a rate nearly half of that reported in comparable summer events such as the 2008 Beijing Olympics (24), 2012 London Olympics (12), 2016 Rio Olympics (11), 2020 Tokyo Olympics (13), and 2018 Summer Youth Olympics (25). This variance may stem from differences in event programming or intensity. It’s also possible that this study’s data, limited to visits to the polyclinic in the athletes’ village, did not capture milder injuries without need for treatment to polyclinic. Nonetheless, competitive sports injuries often require comprehensive medical attention despite initial on-site and team medical care, making this study’s approach suitable for reflecting the injury and illness patterns among athletes at this event. Compared to the 2015 Granada Winter World University Games (20), injury rates were slightly lower, potentially influenced by event characteristics and climate. Few epidemiological studies have focused on young elite athletes in major sporting events; only three studies have addressed injuries among young elite athletes at the Winter Youth Olympics (26–28), and one study reported on injuries at the 2018 Summer Youth Olympics (25). This research contributes new insights into athlete protection and event management during large-scale events, including the World University Games.

Our research findings indicate that injuries among athletes at the 2021 Summer World University Games involved 540 anatomical sites, with lower extremities (thighs, lower legs, knees, and ankles) being the most affected, accounting for 56.1% of all injury locations. Specifically, injuries to the thigh (*n* = 119), lower leg (*n* = 84), and knee (*n* = 66) were most prevalent. This aligns with previous studies on summer sporting events; for instance, the 2016 Rio Olympics reported thigh (*n* = 108), lower leg (*n* = 90), and knee injuries (*n* = 130) (11), and the 2020 Tokyo Olympics noted knee injuries (*n* = 114) and thigh injuries (*n* = 89) (13). In contrast, the patterns differed from the 2015 Granada Winter World University Games (head, shoulder, and right hand injuries most common) (20) and the 2022 Beijing Winter Olympics (knee, head, shoulder, waist, and hand injuries) (14). The predominance of lower limb injuries in Chengdu suggests possible differences in event characteristics between winter and summer games. Based on this, proactive and targeted interventions for lower limb injuries during pre-event training may reduce the likelihood of most injuries and mitigate severe consequences. As the four most commonly reported injury mechanisms were overuse, non-contact trauma, contact with another athlete and contact with a stationary object (12), monitoring training loads by coaches and athletes, combined with neuromuscular training, the use of protective equipment, and environmental optimization, can effectively reduce the risk of injuries. Approximately 36.2% of athletes sustained injuries in two or more anatomical locations (*n* = 114), with 38.0% of male athletes (*n* = 65) and 34.0% of female athletes (*n* = 49) affected in two or more areas, significantly impacting both genders similarly to previous reports (11, 12). This may be due to a combination of physiology, sport characteristics and protective measures. Even minor injuries during major sporting events can restrict athletes’ participation and hinder performance potential, thus impeding achieving optimal results. Targeted protection and gender-specific training are key to reducing injury risk. Therefore, sports teams should enhance injury prevention and management strategies tailored to the specific gender and event characteristics of athletes.

Similar to other major sporting events, training or competition is the primary cause of injuries. The distribution of injuries during the 2021 Summer World University Games (45.1% vs. 39.7%) mirrors that reported in the 2012 London Olympics and the 2016 Rio Olympics, but differs from the proportions reported in the 2008 Beijing Olympics (72.6% vs. 26.2%) (11, 12, 24). This study identified 15.2% of injuries unrelated to sports or training, including 41 new injuries and 7 old injuries, highlighting the need for athletes and teams to enhance awareness and prevention of non-combat injuries in new environments. Although strict training was conducted before the competition, there were still a few minor injuries whose causes have not been accurately recorded by doctors. This may lead to an underestimation of competition and training-related injuries in this World University Games. Most injuries at the 2021 Summer World University Games were new injuries (248 individuals), equivalent to 3.8 new injuries per 100 athletes, with only 13 cases of recurrent old injuries and 6 cases requiring ongoing treatment for old injuries. Although similar distributions have been reported in previous Summer and Winter Olympics, limitations in recording specific injury mechanisms underscore the need for cautious interpretation of these figures.

### 4.3 Illness risk in the world university games

Considering the global situation of the COVID-19 pandemic, the 2021 Summer World University Games were postponed for 2 years to ensure the safety and health of all involved, including athletes. From a healthcare perspective, Chengdu has conducted a series of emergency drills for infectious disease prevention and control, establishing a robust medical support team. This effort has been acclaimed by international medical officials of FISU as the best prepared University Games in terms of medical readiness among all previous editions. Because the global novel coronavirus pneumonia had returned to normal production and life order, the global epidemic was still reported from time to time, the polyclinic was highly vigilant for the prevention and control of infectious diseases, and the setting of the triage desk was highly consistent with the setting of the polyclinic of the Tokyo Olympic Athletes’ Village (29), which greatly improved the ability to identify infectious diseases early, provided new case support for the triage of the polyclinic of the athletes’ village. Our research findings indicate the effectiveness of the measures implemented. The incidence of illness among athletes in the 2021 Summer World University Games was 2.5%, significantly lower than that reported for the 2016 Rio Olympics and the 2020 Tokyo Olympics (11, 13), as well as the 2018 Summer Youth Olympics (25). However, it was three times higher than the illness rate at the 2015 Winter Universiade (20), which may be attributed to factors such as the climate and dietary habits of the host city.

Importantly, the 2021 Summer World University Games did not experience the anticipated infection issues, and timely and accurately detected and dealt with novel coronavirus pneumonia, malaria, chikungunya, and cryptococcal intestinal infection etc. imported infectious diseases. This shows that the infectious disease prevention and control measures formulated through in-depth research and strictly implemented before this university games was successful. The main framework taken include as following. First, specialized trainings for polyclinic staffs on the prevention and control of infectious diseases have been conducted, and standardized clinical guidelines for prevention and control were provided for reference. Second, a triage desk was established in outpatient departments, focusing on initial case screening based on clinical symptoms (e.g., fever, diarrhea) and epidemiological history. Third, independent infectious disease treatment unit was set up and a closed-loop management of suspected cases under the guidance of specialists has been implemented. Finally, a strict two-way isolation system for personnel and materials have been enforced between the infectious disease treatment areas and regular medical areas to eliminate the risk of cross-infection.

Similar to previous studies on major summer sports events (11, 12, 30–32), athletes at the 2021 Summer World University Games were predominantly affected by respiratory and digestive system issues, with female athletes experiencing a higher incidence of illness compared to males. This may be due to sex-based differences in immune responses (e.g., estrogen enhances immune function but may increase inflammatory reactions) and women’s higher propensity to report health issues. This underscores the importance of designing and implementing targeted disease prevention programs. Meta-analyses have shown that injuries and illnesses significantly increase the risk of athletic failure and adversely affect final rankings, both directly and indirectly (6). Therefore, injury and illness prevention strategies must be a focal point for success-oriented athletes or teams. Recommendations include, but are not limited to: Educating on personal hygiene and prevention strategies to stop spreading the infectious organism to the surrounding team and other athletes (33, 34); Providing dedicated health support for women; Distributing heat acclimatization guidelines and implementing heat stroke prevention measures.

### 4.4 Limitations

Our study’s strength lies in the accurate documentation of athlete injuries and illnesses through electronic medical records in the athletes’ village, thereby enhancing the precision of reporting for athlete visits due to injuries and illnesses. However, our research is limited by the lack of venue-specific medical records data. This limitation is akin to previous epidemiological surveys conducted during Olympics, where data collection involving various collectors from different backgrounds and multiple venues introduced certain systematic biases, thereby limiting the accuracy of the reports (35). This study only analyzes the medical treatment of athletes in the athletes’ village, which cannot represent the medical services of the entire 2021 Summer World University Games. Because this study was a retrospective analysis rather than a prospective study, there was no quality control on whether doctors accurately record the cause of injury in medical records, which may lead to an underestimation of the training and competition-related injuries of athletes in this college sports meeting. This provides a reference for subsequent studies on large-scale sports competitions using electronic medical records of hospital information systems.

All athletes have access to healthcare through the polyclinic at the athletes’ village. However, less severe injuries (which may not always require medical attention) could be overlooked (36), and the availability of medical teams from various delegations means not all athletes necessarily visit the village clinic. This situation could potentially lead to some bias in injury and illness records. This study is a retrospective case analysis based on medical records after the games, and there is no relevant professional questionnaire recording and analysis of the causes of injuries of athletes (37), so it is impossible to conduct an in-depth analysis of the causes of injuries.

### 4.5 Practical implications

The accumulating epidemiological evidence from major sporting events indicates a higher risk of injuries (11–13, 26, 27, 38). To mitigate these risks, more precise data related to specific risk factors and mechanisms are needed. While our data only encompass injury and illness occurrences among athletes in the 2021 Summer World University Games athletes’ village, our study represents the first to provide injury rates specific to this event. This not only aids in formulating preventive measures for athletes and coaches but also serves as a reference and advisory resource for medical personnel and event organizers in planning healthcare services. Importantly, it provides scientific grounds for organizing and reducing risks in international university sports events. Additionally, our research offers insights into athlete clinic access and timing, which can further enhance medical service provisions, refine athletes’ village management, improve athlete satisfaction and participation experiences, thereby elevating overall event quality and reputation, and enhancing its appeal and impact.

Systematic monitoring of injuries and illnesses will aid in early identification of at-risk sports and athletes (2), Collecting such data will allow the development injury risk predictive profile for each athlete in each sport, furthermore, a follow-up survey of injured athletes will provide some insight into the long-term impact of injuries beyond the time of the tournament (39), maximizing the protection of elite athletes’ health (40). Therefore, we advocate for the International University Sports Federation to establish an interdisciplinary team and a scientifically rigorous monitoring system, ensuring that university athletes minimize risks of physical injuries, illnesses, or psychological harm during training and competition.

## 5 Conclusion

In summary, 4.9% of the athletes were injured and 2.5% ill during the 2021 Summer World University Games in Athletes’ Village. The differences in injury severity and illness rates among athletes of different genders, along with the higher proportion of lower limb injuries, provide valuable insights for athlete protection. In the medical support of the athlete village in large-scale sports events, it is necessary to consider increasing the MRI configuration requirements to better meet the diagnosis and treatment needs of athletes’ injuries within the village. The athlete injuries in large-scale sports events can be studied through the use of electronic medical records of the hospital information system of the comprehensive clinic in the athlete village, but prospective quality control of the injury causes recorded by doctors in the medical records is required. Taking appropriate prevention and control measures for infectious diseases can effectively prevent the prevalence of infectious diseases during large international sports events. Our findings can inform future planning and medical care for large-scale competitions, and lay the groundwork for further research into risk factors and injury mechanisms within this cohort.

## Data Availability

The raw data supporting the conclusions of this article will be made available by the authors, without undue reservation.
